# Autonomic reinnervation and functional regeneration in autologous transplanted submandibular glands in patients with severe keratoconjunctivitis sicca

**DOI:** 10.1038/s41368-018-0014-1

**Published:** 2018-04-25

**Authors:** Xueming Zhang, Ningyan Yang, Xiaojing Liu, Jiazeng Su, Xin Cong, Liling Wu, Yan Zhang, Guangyan Yu

**Affiliations:** 1 Center for Salivary Gland Diseases and Department of Oral and Maxillofacial Surgery, Peking University School and Hospital of Stomatology, Beijing, China; 2 Department of Pediatric Dentistry, Beijing Stomatological Hospital and School of Stomatology, Capital Medical University, Beijing, China; 3 Department of Physiology and Pathophysiology, Peking University School of Basic Medical Sciences, and Key Laboratory of Molecular Cardiovascular Sciences, Ministry of Education, Beijing Key Laboratory of Cardiovascular Receptors Research, Beijing, China

## Abstract

Autologous submandibular gland (SMG) transplantation has been proved to ameliorate the discomforts in patients with severe keratoconjunctivitis sicca. The transplanted glands underwent a hypofunctional period and then restored secretion spontaneously. This study aims to investigate whether autonomic nerves reinnervate the grafts and contribute to the functional recovery, and further determine the origin of these nerves. Parts of the transplanted SMGs were collected from the epiphora patients, and a rabbit SMG transplantation model was established to fulfill the serial observation on the transplanted glands with time. The results showed that autonomic nerves distributed in the transplanted SMGs and parasympathetic ganglionic cells were observed in the stroma of the glands. Low-dense and unevenly distributed cholinergic axons, severe acinar atrophy and fibrosis were visible in the patients’ glands 4–6 months post-transplantation, whereas the cholinergic axon density and acinar area were increased with time. The acinar area or the secretory flow rate of the transplanted glands was statistically correlated with the cholinergic axon density in the rabbit model, respectively. Meanwhile, large cholinergic nerve trunks were found to locate in the temporal fascia lower to the gland, and sympathetic plexus concomitant with the arteries was observed both in the adjacent fascia and in the stroma of the glands. In summary, the transplanted SMGs are reinnervated by autonomic nerves and the cholinergic nerves play a role in the morphological and functional restoration of the glands. Moreover, these autonomic nerves might originate from the auriculotemporal nerve and the sympathetic plexus around the supplying arteries.

## Stomatology: Nerve Supply to Transplanted Glands in Dry Eye Syndrome

Regenerated nerves play a role in restoring the function of transplanted submandibular glands (SMGs) in treating dry eye syndrome. Dry eye syndrome, or keratoconjunctivitis sicca (KCS), is a leading cause of patients visiting ophthalmologists, and one effective treatment in severe cases is transplanting SMGs (to the temporal area). Autonomic nerves have an important function in maintaining the secretory function of salivary glands, and a team headed by Yan Zhang and Guang-Yan Yu at Peking University, China investigated whether those nerves underwent reinnervation (restoration of nerve control) with transplanted SMGs in KCS patients and in a rabbit model. The authors found that in both the patients and rabbit model, the transplanted SMGs were reinnervated and the regenerated nerves played a role in restoring the glands’ function. The findings offer new insights into managing SMG transplantation.

## Introduction

Keratoconjunctivitis sicca (KCS), also known as dry eye syndrome, is one of the leading causes for patients visiting ophthalmologists.^1,2^ Microvascular autologous submandibular gland (SMG) transplantation has been proved to be an effective treatment for severe cases of KCS.^3–10^ The clinical time-course after SMG transplantation could be divided as follows: (1) 1st–2nd day of transient hypofunctional period; (2) 3rd–6th day of temporary epiphora period; (3) 3 months of latent period; (4) recovery period thereafter.^3^ The major complications that may cause transplantation failure are vascular crisis in the initial phase and chronic obstructive sialadenitis during the latent period when hyposecretion lasts.^11,12^ Administration of capsaicin and carbachol have been introduced to deal with these problems.^11,13^ After the latent period, the transplanted glands restore the secretory function spontaneously. However, excessive saliva tear secretion, or termed as epiphora, occurs in almost half of the patients 6 months after transplantation, especially when they are in a high-temperature environment or doing physical activities.^14^ Surgical reduction of the glands is usually needed to reduce the secretion. In order to find better ways to prevent or treat such complications, the regulatory mechanism of the transplanted glands need to be clarified.

Autonomic nerves play an important role in maintaining the secretory function of salivary glands. Besides, the parasympathetic stimulation has been proved to improve the epithelial regeneration after irradiation injury.^15^ However, the transplantation procedure left the glands denervated. In view of the significant role of the autonomic nerves, the restoration of secretory function and even hypersecretion in the recovery period suggests a possibility of autonomic reinnervation in the transplanted glands. Geerling et al. once reported the appearance of cholinergic and adrenergic nerve fibers in the glands collected from the patients who underwent the reduction surgery.^16^ Nevertheless, the function and the origin of the redistributed nerves are not well clarified. Therefore, in this study, we tried to evaluate the function and the origin of the reinnervated nerves based on the morphological evidences collected from the biopsies of epiphora patients as well as from the rabbit SMG transplantation model.

## Results

### The autonomic nerve redistribution and the morphological change in the parenchyma of human transplanted glands

**Fig. 1:**
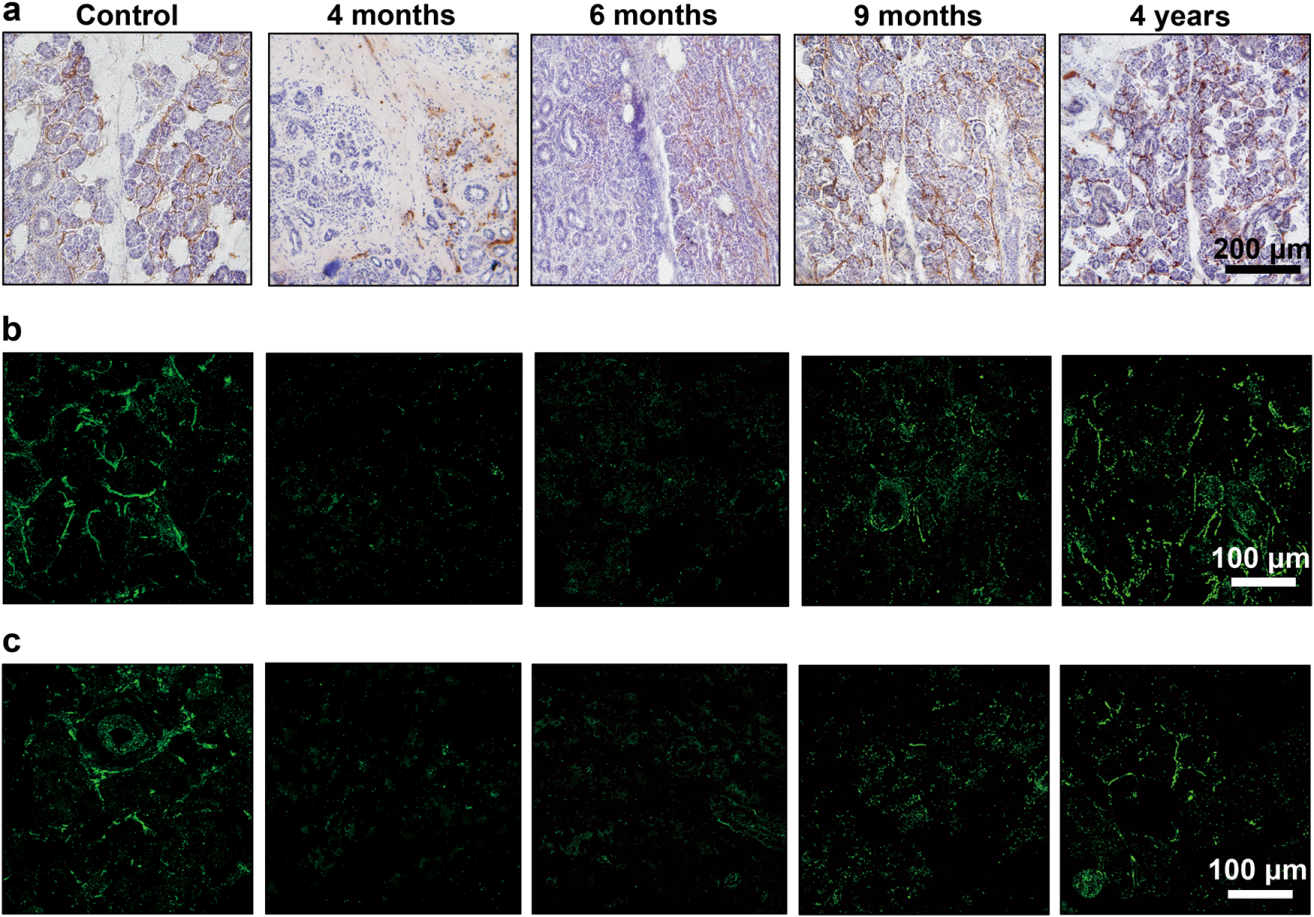
The autonomic nerve redistribution in the parenchyma of the transplanted glands in patients. **a** The cholinergic axons are absent in some lobules in glands 4–6 months post-transplantation but are fully distributed among lobules in the long-term glands (acetylcholinesterase staining). Bar: 200 µm. **b**, **c** Cholinergic (labeled by VAChT) and adrenergic axons (labeled by TH) are scarce in glands 4–6 months post-transplantation and are abundant in the long-term glands (immunofluorescence). Bar: 100 µm. VAChT vesicular acetylcholine transporter, TH tyrosine hydroxylase

**Fig. 2:**
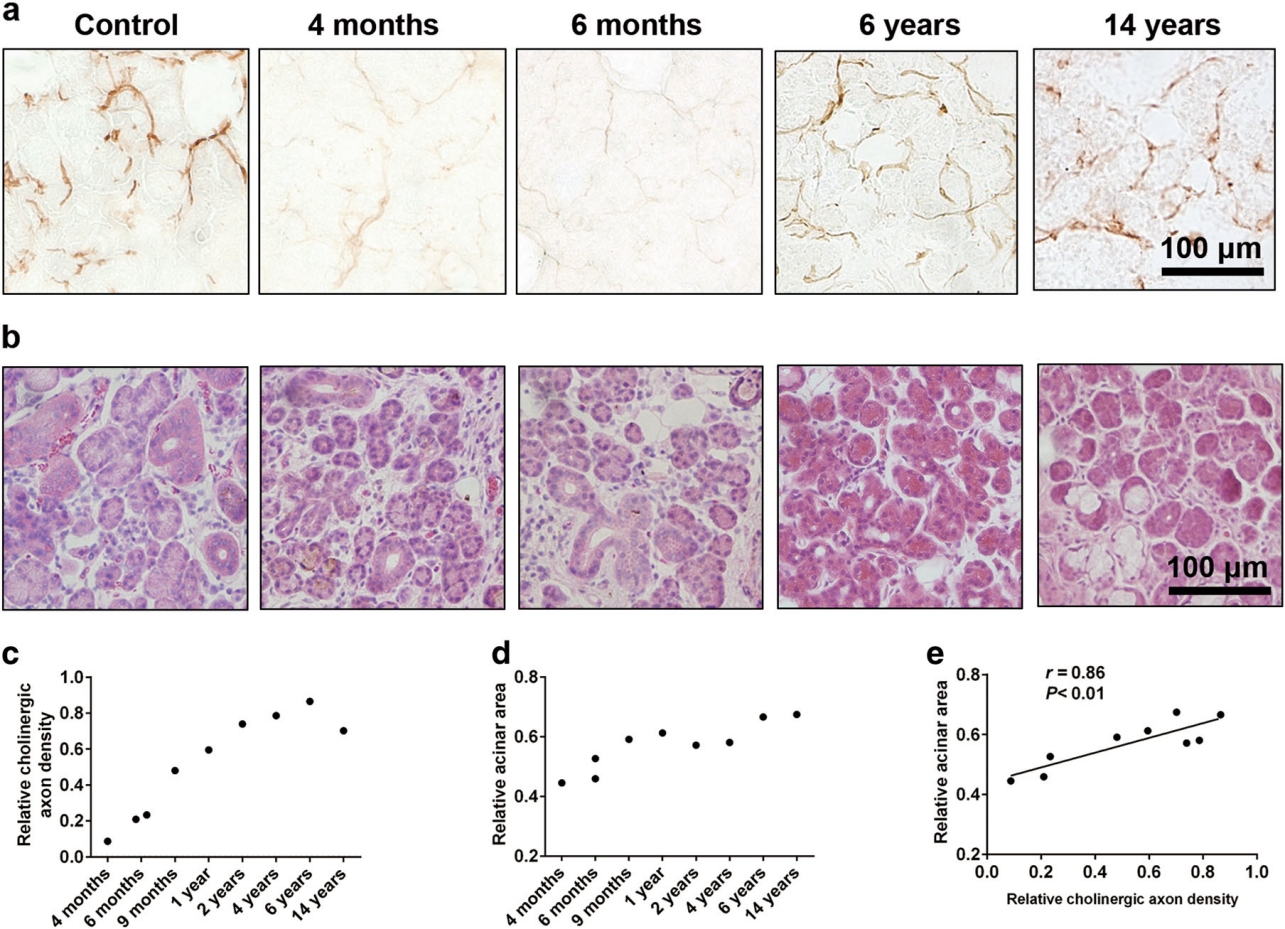
The time-dependent variation of the cholinergic axon density and the acinar area of the transplanted glands in the patients. **a** Cholinergic nerve density is increased in the long-term cases compared with the cases within 6 months post-transplantation (acetylcholinesterase staining). Bar: 100 µm. **b** Severe atrophy and fibrosis are detected in cases within 6 months post-transplantation and were relieved in the long term (hematoxylin-eosin staining). Bar: 100 µm. **c**, **d** The cholinergic axon density (**c**) and the acinar area (**d**) in the parenchyma of each case are quantitatively analyzed and the data is expressed by ratio to control. **e** The correlation between the axon density and acinar area is statistically significant (Pearson correlation test, see *r* and *P* values in the figure)

The histological examinations of the 9 transplanted gland specimens derived at different timepoints ranging from 4 months to 14 years revealed a time-dependent variation of nerve distribution and glandular structure. The cholinergic nerves were observed in all cases, but the distribution area and quantity of the nerve fibers were different. As shown by acetylcholinesterase staining, the cholinergic axons were detected in some lobules but absent in others in glands of 4–6 months post-transplantation. Comparatively, the cholinergic axons were found in more lobules in the long-term cases (Fig. 1a). To get a detailed distribution of both cholinergic and adrenergic nerves, the sections were immunolabeled by VAChT or TH respectively.^15^ As shown by immunofluorescence, both cholinergic and adrenergic nerve axons were rare in the parenchyma within 6 months post-transplantation, but increased in number in the longer cases (Fig. 1b, c). Histological staining suggested that the variation of the cholinergic axon density and the acinar area was time dependent (Fig. 2a–d). When the nerve axon density was low at 4 or 6 months post-transplantation, severe parenchyma atrophy and fibrosis were observed in these 3 cases, of which the acinar area was merely 47.7% of the control glands. However, the cholinergic axon density and the acinar area showed an increased tendency in the long-term cases. The cholinergic axon density correlated with the acinar area (*r* = 0.86, *P* < 0.01, Fig. 2e).

### The cholinergic nerve redistribution, histological change and the secretory function of the glands in the rabbit SMG transplantation model

**Fig. 3:**
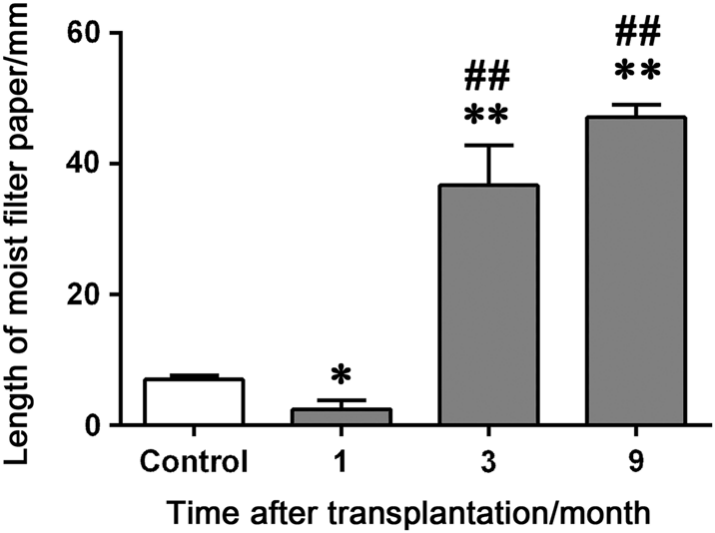
Saliva flow rate of the transplanted glands in rabbits. Saliva flow rate are compared among different post-operative time point by one-way ANOVA. Error bars represent means ± SEM for *n* = 6 in control, 1 month and 3 months group, and *n* = 4 in 9 months group. *P < 0.05, **P < 0.01, compared with controls; ^##^*P* < 0.01, compared with 1 month

As there were few opportunities to get a series of SMG biopsies from the patients who underwent the transplantation surgery, we established a rabbit SMG transplantation model. The secretion of the transplanted gland was significantly decreased at rest condition at 1 month post-transplantation (*P* < 0.05), but increased obviously at 3 and 9 months post-transplantation (*P* < 0.01, Fig. 3). This salivation change was coincident with the clinical observation that transplanted glands underwent different stages, including the latent period and the recovery period.

**Fig. 4:**
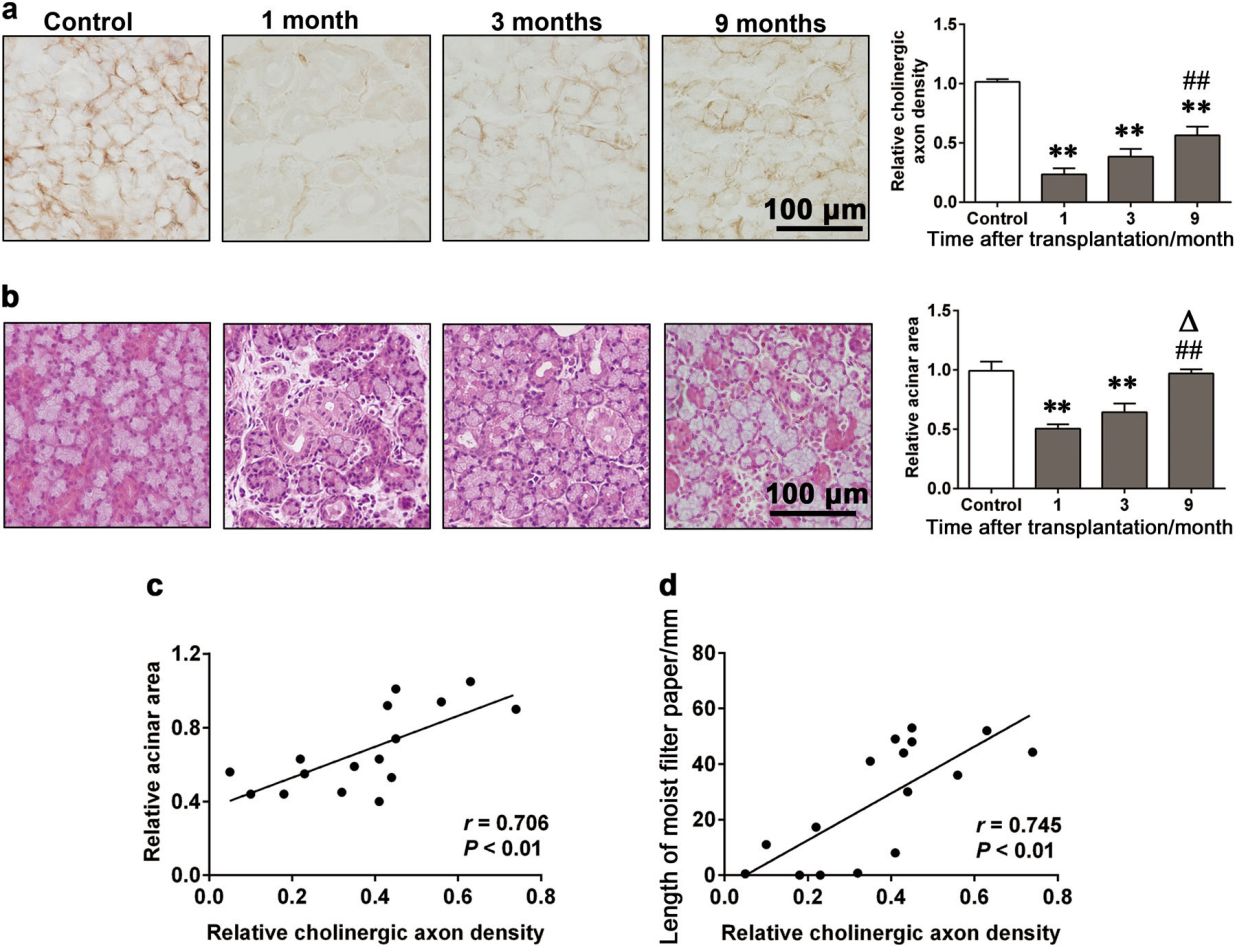
The time-dependent variation of the cholinergic axon density and its relationship with acinar area and secretion function of the transplanted glands in rabbits. **a** Cholinergic nerve fibers in parenchyma of the grafts at different time-points (acetylcholinesterase staining). Bar: 100 µm. **b** Histological structures of the grafts at different time-points (hematoxylin-eosin staining). Bar: 100 µm. Relative cholinergic axon density (**a**) and acinar area (**b**) are compared among different post-operative time point by one-way ANOVA. The data are expressed by ratio to control and presented as means ± SEM or *n* = 6 in control, 1 month and 3 months group, and *n* = 4 in 9 months group. **P < 0.01, compared with controls; ^##^*P* < 0.01, compared with 1 month; ^Δ^*P* < 0.05, compared with 3 months. **c** The correlation between the cholinergic axon density and the acinar area is significant. **d** The correlation between the cholinergic axon density and the flow rate is significant (Pearson correlation test, see *r* and *P* values in the figures)

The histological staining results showed that there was also a time-dependent variation of the cholinergic axon density and the acinar area. Although the cholinergic axon density was decreased at all time-points after transplantation (*P* < 0.01), the nerve density at 9 months post-transplantation was higher than that at 1 or 3 months post-transplantation (*P* < 0.01, Fig. 4a). In the meantime, serious gland atrophy and fibrosis were observed at 1 month post-transplantation, whereas the mild restoration of glandular structure occurred at 3 months post-transplantation and the structural restoration further improved at 9 months post-transplantation. The acinar area decreased at 1 and 3 months post-transplantation (*P* < 0.01), but increased later at 9 months post-transplantation (*P* < 0.05) and restored to the control level (Fig. 4b). The cholinergic axon density correlated with the acinar area (*r* = 0.706, *P* < 0.01, Fig. 4c), and the secretory flow rate of the transplanted glands (*r* = 0.745, *P* < 0.01, Fig. 4d).

### The autonomic nerve distribution pattern in the temporal fascia and the stromal area of the transplanted glands

**Fig. 5:**
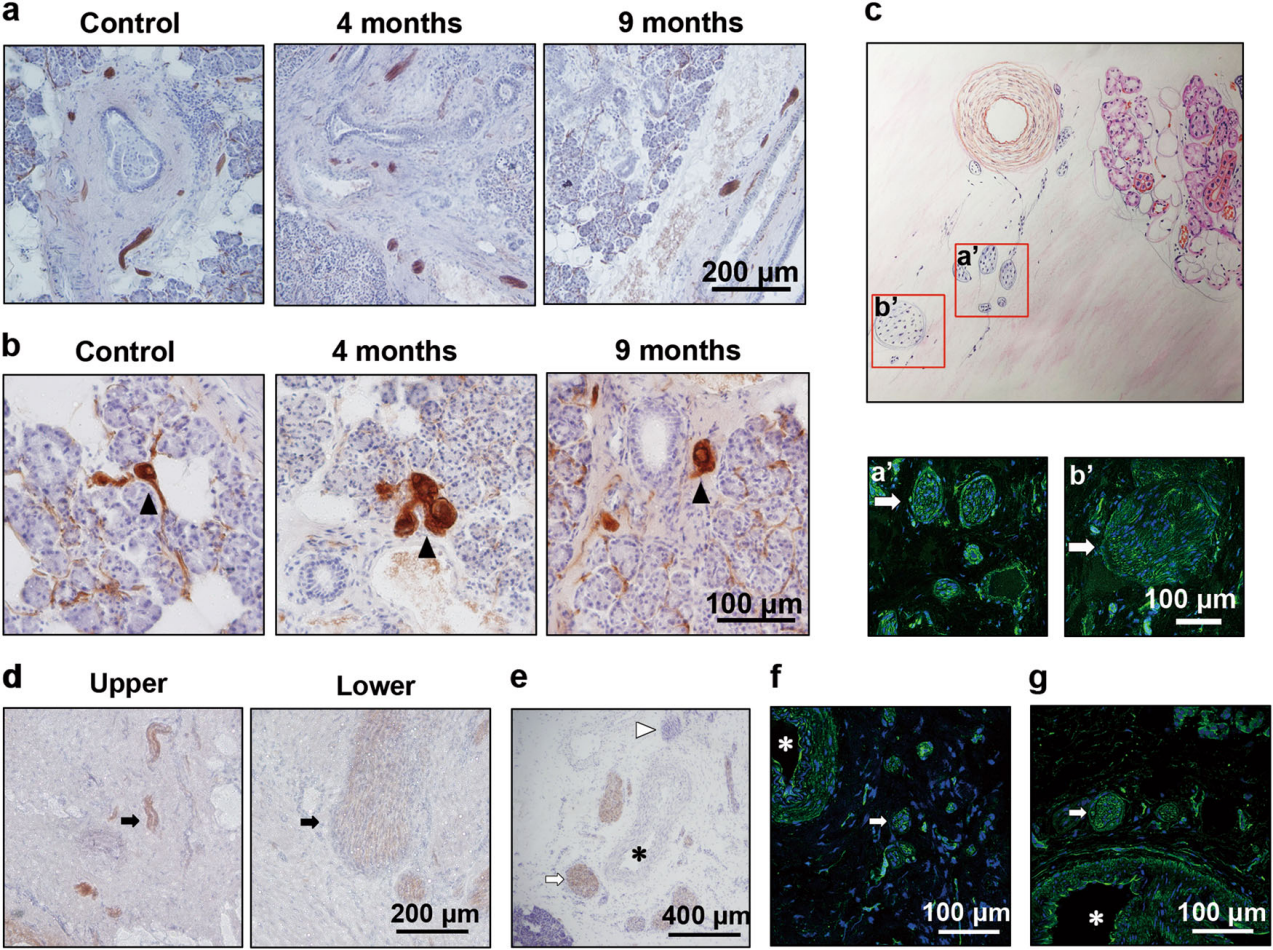
The autonomic distribution pattern in the temporal fascia and the stromal area of the glands in the patients. **a** Cholinergic nerve bundles are shown in the stromal area of the transplanted glands (acetylcholinesterase staining). Bar: 200 µm. **b** Arrowheads show the ganglionic cells (acetylcholinesterase staining). Bar: 100 µm. **c** Adrenergic nerve bundles (labeled by TH) of different diameter in the temporal fascia. Two views (a′, b′) with different distance to the gland are illustrated in the drawing picture (immunofluorescence). Bar: 100 µm. **d** Cholinergic nerve trunks are only located in the fascia lower to the gland rather than in the upper (acetylcholinesterase staining). Bar: 200 µm. **e** Cholinergic (arrow) and non-cholinergic (arrowhead) nerve bundles scattered around the arteries (asterisk) that supply the glands (acetylcholinesterase staining). Bar: 400 µm. **f**, **g** Adrenergic (**f**, labeled by TH) and cholinergic (**g**, labeled by VAChT) nerve bundles (arrow) are concomitant with the arteries (asterisk) in the stromal area of the gland (immunofluorescence). Bar: 100 µm

To determine the origin of the reinnervated autonomic nerves, we explored the nerve distribution pattern in the temporal fascia and the stromal area of the transplanted glands. Although the cholinergic axon density was low and the nerve fibers were unevenly distributed among lobules in the glands of patients removed within 6 months post-transplantation, the cholinergic nerve bundles and the ganglionic cells were constantly detected in the stromal area of all cases (Fig. 5a, b). Both adrenergic and cholinergic nerve bundles were observed to exist in the temporal fascia adjacent to the glands. As shown in one case, the diameter of the adrenergic nerve truck emerging in the fascia distal to the gland was around 200 μm, whereas it was decreased to about 40 μm when the nerve trunk ramified into several branches and made its way close to the gland (Fig. 5c). Similar image was also captured for the cholinergic nerve bundles, in which the diameter was smaller when they were getting closer to the gland parenchyma. Since the samples had been divided into two parts during collection, thicker cholinergic nerve bundles of ~200 μm in diameter were detected in the lower fascia of the gland, while some finer bundles were found to be scattered in the upper part (Fig. 5d). Arteries of 300–800 μm wide were routinely found at the edge of the glands, around which the sympathetic plexus composed of adrenergic or cholinergic axons excited (Fig. 5e). Adrenergic and cholinergic nerve bundles were also detected around the arteries within the stromal area of the glands (Fig. 5f, g).

## Discussion

Autonomic innervation is known to be pivotal in maintaining the secretory function of the salivary glands. In this study, the reinnervation of autonomic nerves was verified in the transplanted SMGs and played a role in the functional recovery in the long term. Meanwhile, the possible origins of the reinnervated nerves were proposed.

Spontaneous autonomic reinnervation occurs in many visceral organ grafts,^17–20^ which seems to be a common pathophysiological phenomenon following organ transplantation. Factors that affect the autonomic reinnervation include the observation time after surgery, the age and systemic conditions of the patients,^21^ the surgical procedure,^18,20,22^ and the application of neurotrophic factors.^15^ Impaired innervation to salivary glands is reported in patients with Sjogren’s syndrome,^23^ diabetes,^24^ and radiation-induced xerostomia.^15^ The leading etiology of the enrolled patients who received SMG transplantation in this study is Stevens–Johnson syndrome, which was characterized as a cell-mediated hypersensitivity reaction targeting skin, mucosa and exocrine glands, and no nerve system impairment has been reported.^25^ Thus, autonomic reinnervation was expected to occur spontaneously in these glands. Our results showed that autonomic nerves were present in the transplanted SMGs as early as 4 months after transplantation and increased over time, which suggested that the autonomic reinnervation occurs in the transplanted SMGs.

Previous research suggested that cholinergic signaling promotes gland regeneration after irradiation damage^15^ and reinnervation ameliorates the gland atrophy and dysfunction caused by parasympathectomy.^26–28^ Our results showed the cholinergic axon density and the acinar area of the transplanted glands increased in human transplanted glands over observed time. The cholinergic axon density correlated with the acinar area and the secretory flow rate in the rabbit SMG transplantation model. These findings indicated that the cholinergic reinnervation might promote the functional restoration of the transplanted glands. It is noteworthy that the relative cholinergic axon density and acinar area were smaller than control, while saliva flow rate was quite high 3 months after SMG transplantation in rabbits, which suggests that apart from autonomic reinnervation, other factors including the supersensitivity of the cholinergic receptors,^29^ and the alteration in tight junction proteins that regulate fluid transportation through paracellular pathway^30,31^ may play a role in the excessive secretion of the transplanted SMGs. Moreover, botulinum neurotoxin A (BTXA) inhibits acetylcholine release by binding to an acceptor protein on the presynaptic membrane of the acetylcholine nerve terminal.^32^ It has been reported that BTXA was effective in reducing the secretion of the transplanted SMG in patients^33^ and rabbits,^34,35^ which provided additional evidences for the functional reinnervation of the cholinergic nerves in transplanted SMG.

Previous studies suggest that the reinnervation of the grafts is the ingrowth of the nerves from the host tissue.^17,18^ On the basis of our results of the nerve distribution pattern in the temporal fascia and the gland stroma, we deduced that the ganglionic cells originally located in the glands might survive after transplantation and the autonomic axons sprouting from the recipient bed prolong into the grafts and reinnervated by developing functional connections with the survived ganglionic cells or with the glandular epithelium directly. There are two possible origins of reinnervated nerves in the transplanted SMGs, which are as follows: (1) sympathetic plexus around arteries including adrenergic and cholinergic postganglionic fibers. Rich blood supply to the transplanted SMG is usually observed during the reduction surgery, and most patients complain epiphora induced by physical activity or high-temperature environment, which activates the sympathetic nervous system. These observations support this hypothesis. (2) parasympathetic fibers contained in the auriculotemporal nerve. The auriculotemporal nerve, which rules the secretion of parotid gland, is usually exposed in the recipient bed during the transplantation surgery, and its location anatomically correspond to our results that large cholinergic nerve trunks were observed in the temporal fascia lower to the gland. Our previous work has demonstrated acid stimulated secretion in some epiphora patients’ glands with proper cholinergic innervation by ^99m^Tc scintigraphy,^36^ which backs up the origin from auriculotemporal nerve since there exits an olfactory salivary reflex in these cases.

In summary, we confirmed that the transplanted glands are reinnervated both in the patients and in the rabbit model, and the regenerated cholinergic nerves play a role in the morphological and functional restoration of the transplanted SMGs. Moreover, these autonomic nerves might originate from the auriculotemporal nerve and the sympathetic plexus around the supplying arteries. These findings enlighten us with new insights in the management of SMG transplantation.

## Materials and methods

### Patients and specimen collection

**Fig. 6:**
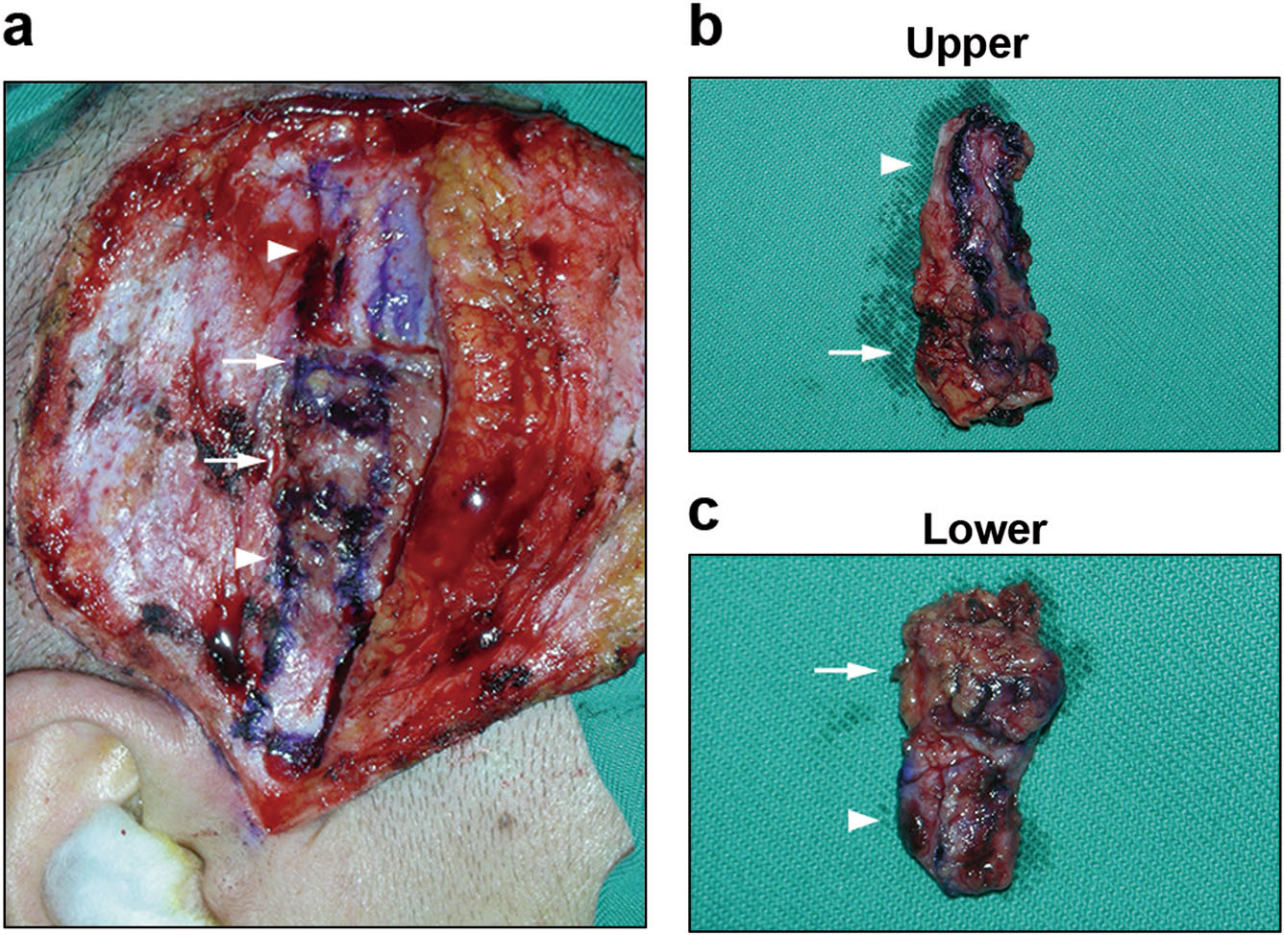
Sample collection. The reduced gland (arrows) was removed with the attached temporal fascia (arrowheads) together (**a**) and divided into the upper part (**b**) and the lower part (**c**)

Nine KCS patients (all female; median age 41 years, range from 22 to 62 years) who underwent surgical reduction of the grafts between July 2015 and December 2016 due to severe epiphora after autologous SMG transplantation were enrolled in this study. The etiology of KCS was Stevens–Johnson syndrome in 6 patients, chronic keratoconjunctivitis in 2 and unknown in 1. The time interval between the initial transplantation and secondary surgical reduction of the 9 cases was 4 months, 6 months, 6 months, 9 months, 1 year, 2 years, 4 years, 6 years, and 14 years, respectively. During the initial autologous SMG transplantation surgery, the glands were dissected from the submandibular area and transplanted to the temporal area with revascularization, and the opening of the Wharton’s ducts were sutured into the upper conjunctival fornixes as described previously.^3^ The secretion of transplanted SMGs was used as a substitution for tear. Later, the surgical reduction was performed to control the complication of epiphora when necessary. Briefly, the original approach of the initial transplantation surgery was followed. Partial gland together with the surrounding temporal fascia was removed and used as the biopsy sample, which was divided into the upper and the lower parts for histological examinations (as shown in Fig. 6). Five SMG controls were collected from patients who underwent functional neck dissection for primary tongue squamous carcinoma and were histologically confirmed normal by a pathologist. The study was approved by the Ethics Committee for Human Experiments of Peking University (No. IRB00001052-08048). All patients had signed on the informed consent form.

### Histochemical staining

Tissues were frozen and cut into 20 μm-thick sections. The section plane involves both the gland tissue and the adjacent temporal fascia. The slices were stained for acetylcholinesterase activity according to the protocol described previously.^37^ Briefly, the specimens were incubated in acetylthiocholine iodide (Sigma, St. Louis, MO, USA) in the presence of tetraisopropyl pyrophosphoramide (3 × 10^6^ mol·L^−1^) to inhibit nonspecific cholinesterase. After 1 h incubation at 37 °C, the slices were counterstained with hematoxylin or mounted without counterstaining for quantitative analysis. Samples were also fixed in 4% paraformaldehyde and embedded with paraffin, sectioned into slices (5 μm) and stained with haematoxylin and eosin (H&E). Images were captured under a light microscope (Q550CW, Leica, Manheim, Germany).

For quantitative analysis, the area of 30 randomly selected acini from 6 views of H&E sections was delineated. The positive brown area in the gland parenchyma from 6 randomly selected views of the acetylcholinesterase staining slices was determined. The mean acinar area and the cholinergic axon density were quantified by Image J Software (National Institutes of Health, MD), and the results were presented as ratio to control.

### Immunofluorescence

Frozen sections were blocked with 1% BSA for 2 h, incubated with anti-vesicular acetylcholine transporter (VAChT) antibody (ab68984, Abcam, Cambridge, UK, dilution 1:300), anti-tyrosine hydroxylase (TH) antibody (ab6211, Abcam, Cambridge, UK, dilution 1:500) at 4 °C overnight, and then incubated with AlexaFluor488-conjugated secondary antibody at 37 °C for 1 h. Nuclei were stained with DAPI. Confocal images of 10 μm Z-projection were captured by a laser scanning confocal microscope (Leica TCS SP8, Wetzlar, Germany).

### Rabbit model of SMG transplantation

Twenty-two healthy male New Zealand rabbits weighing 2.3–2.5 kg were used to establish SMG transplantation model. Autologous SMG transplantation was performed as described previously.^30^ Briefly, rabbits were anesthetized with sodium pentobarbital (20 mg·kg^−1^ body weight). The SMGs along with the Wharton’s duct and the supplying vessels were dissected and transplanted to the temporal region, recirculating the gland by anastomosis to the distal end of the external carotid artery and the temporary vein. The Wharton’s duct was passed through the subcutaneous tunnel and the duct opening was sutured to the lower fornix of the eyelid. A polyethylene catheter was inserted into the duct and indwelt for 7 days. Normal saline irrigation through the catheter was performed to prevent duct obstruction. The rabbits were killed at 1 month (*n* = 6), 3 months (*n* = 6) and 9 months (*n* = 4) post-operation and the grafts were removed for histological examinations. Six sham-operated rabbits were served as controls. All experimental procedures were approved by the Ethics Committee of Animal Research, Peking University Health Science Center and were in accordance with the Guidance of the Ministry of Public Health for the care and use of laboratory animals.

### Measurement of salivary secretion

One day before killing, the rabbits were anesthetized and the catheters were inserted into the Warton’s ducts. The secretory volume was measured in conscious animals under rest condition by modified Schirmer’s test between 9:00 a.m. and 10:00 a.m. The length of filter paper (35 mm × 5 mm) moistened by the saliva-tear flowing out of the catheter in 5 min was recorded.^38^ Each collection was repeated for 3 times, and the average values were calculated.

### Statistical analysis

The data were presented as means ± SEM. Differences for multiple groups were analyzed by using one-way ANOVA followed by Bonferroni test. The correlations between the acinar area and the cholinergic axon density were analyzed by Pearson correlation test. All the data were analyzed by using GraphPad software (GraphPad Prism 5, CA, USA). *P* < 0.05 was considered to be statistically significant.
